# Superfoods for Type 2 Diabetes: A Narrative Review and Proposal for New International Recommendations

**DOI:** 10.3390/medicina59071184

**Published:** 2023-06-21

**Authors:** Carla Pires

**Affiliations:** CBIOS—Research Center for Biosciences & Health Technologies, Universidade Lusófona, Campo Grande 376, 1749-024 Lisbon, Portugal; p5558@ulusofona.pt

**Keywords:** T2DM, superfoods, HbA1c, metabolic control, glycaemia, new recommendations/guidance

## Abstract

*Background and Objectives*: Type 2 diabetes mellitus (T2DM) is a chronic metabolic disease affecting an estimated 537 million individuals worldwide. ‘Superfoods’ can be integrated into the diet of T2DM patients due to their health benefits. Study Objectives: (i) To carry out a narrative review of ‘superfoods’ with the potential to reduce glycaemic levels in T2DM patients (2019 to 2022), (ii) to identify ‘superfoods’ with the potential to reduce HbA1c and (iii) to propose new guidance on the use of ‘superfoods’. *Materials and Methods*: A narrative review was carried out using the databases PubMed, SciELO, DOAJ and Google Scholar. The keywords were [“type 2 diabetes” and (“food” or “diet” or “nutrition”) and (“glycaemia” or “glycemia”)]. Only review studies were included. *Results*: Thirty reviews were selected. The ‘superfoods’ identified as having a potential impact on glycaemic control were foods with polyphenols (e.g., berries), fermented dairy products, whole cereals/grains, nuts and proteins, among others. The possibility of an extensive reduction in Hb1Ac was reported for fermented dairy products, especially yoghurts enriched with vitamin D or probiotics (HbA1c reduction of around 1%) or by increasing the fibre intake by 15 g (or up to 35 g) (HbA1c reduction of around 2%). *Conclusion*: It is recommended that the identified ‘superfoods’ are included in the diet of T2DM patients, although this should not substitute an appropriate diet and exercise plan. In particular, yoghurts and an increased fibre intake (by 15 g or up to 35 g) can be used as nutraceuticals. New recommendations on the introduction of ‘superfoods’ in the diet of T2DM patients have been proposed.

## 1. Introduction

Type 2 diabetes mellitus (T2DM) is a chronic disease motivated by the body’s ineffective use of insulin [1]. According to the International Diabetes Federation Diabetes Atlas, the number of adults (20–79 years) with diabetes worldwide is predicted to be as follows: 537 million (2021), 643 million (2030) and 784 million (2045), indicating an expected increase of 46% [2]. Blindness, kidney failure, heart attack, stroke and lower limb amputation are among the major complications of diabetes. The prevention or delay in the onset of T2DM can be achieved by a healthy diet, regular physical activity, maintaining a normal body weight and avoiding the use of tobacco [1]. According to the American Diabetes Association, diabetes is diagnosed when the level of glycated haemoglobin A1c (HbA1c) is greater than or equal to 6.5%, the fasting plasma glucose is 126 mg/dL or higher and the oral glucose tolerance test (OGTT) is greater than or equal to 200 mg/dL 2 h after an overnight fast and the administration of a bolus of 75 g oral glucose [3].

In general, the term ‘superfood’ is applied to market foods with significant health benefits, i.e., foods that can prevent diseases, as well as improve overall health, although there is not a consensual definition. These foods tend to be rich in certain nutrients, such as antioxidants or omega-3 fatty acids (e.g., flax, chia, hulled sunflower or hemp seeds) [4]. The American Diabetes Association recommends the consumption of the following ‘superfoods’ in the diet of T2DM patients: (i) beans (e.g., kidney, pinto, navy or black beans), because they provide protein without the saturated fat; (ii) dark green leafy vegetables (e.g., spinach, collards and kale), because they provide vitamins, such as vitamin K, iron, calcium and potassium, and are low in calories and carbohydrates; (iii) citrus fruits, since they contain fibre, vitamin C, folate and potassium; (iv) berries (e.g., blueberries and strawberries) due to their antioxidant activity; (v) tomatoes, due to their vitamin C, vitamin E and potassium content; (vi) fish that is high in omega-3 fatty acids (e.g., salmon, herring, sardines, mackerel, trout and albacore tuna), as omega-3 fats reduce the risk of heart disease and inflammation; (vii) nuts, which contribute to controlling hunger and are generally a good source of omega-3 fats; (viii) whole grains (whole oats, quinoa, whole grain barley and farro), which are good sources of fibre, as well as vitamins and minerals; and (ix) milk and yoghurt (lower in fat and added sugar), which are rich in calcium and vitamin D (enriched products) [5]. ‘Superfoods’ should preferably be integrated into a medical nutrition therapy plan. As an example, the consumption of ‘superfoods’ can have a positive impact on the control of metabolic syndrome parameters, such as waist circumference, body mass index (BMI), blood pressure or the concentrations of high-density lipoprotein (HDL) cholesterol, triacylglycerol or glucose [6].

A successful medical nutrition therapy plan can reduce HbA1c to a similar or greater extent than T2DM medication (up to 2.0% (in T2DM) and up to 1.9% (in type 1 diabetes) at 3–6 months) [7,8]. Mediterranean-style, low-fat or low-carbohydrate diets are the three most relevant and suitable eating plans for the prevention of prediabetes or T2DM [7]. The Mediterranean-style eating pattern is based on the consumption of a lot of vegetables, salad and fruit; moderate portions of whole grain, starchy carbohydrates; more vegetable sources of protein, such as nuts and pulses, as well as fish and poultry; and healthy fats (e.g., olive oil), while red and processed meats, sugars and refined starchy carbohydrates should be reduced [7,8]. Additionally, the Centers for Disease Control and Prevention (CDC) recommend “The Plate Method”, which is based on a nine inch dinner plate: half of the plate should be filled with non-starchy vegetables (e.g., salad, green beans, broccoli, cauliflower, cabbage and carrots); one quarter of the plate should be filled with a lean protein (e.g., chicken, turkey, beans, tofu or eggs); and the other quarter of the plate should be filled with carbohydrates (e.g., starchy vegetables: potatoes and peas), rice, pasta, beans, fruit and yoghurt or milk [9].

In this sense, the aims of this study were (i) to carry out a narrative review of review studies (systematic or meta-analysis reviews, as well as narrative reviews, including commentaries) published between 2019 and 2022 about ‘superfoods’ (or groups of foods) with the potential to reduce glycaemic levels in T2DM patients; (ii) to identify ‘superfoods’ with the potential to reduce HbA1c; and (iii) to propose new guidance regarding the use of the identified ‘superfoods’ in the diet of T2DM patients. Thus, the research questions were as follows:

What are the ‘superfoods’ (or groups of foods) with the potential to control glycaemia in T2DM patients?

What are the ‘superfoods’ (or groups of foods) with the potential to simultaneously control glycaemia plus HbA1c in T2DM patients?

What are the new proposed guidelines regarding the use of the identified ‘superfoods’ in the metabolic control of T2DM patients?

## 2. Materials and Methods

### 2.1. Inclusion and Exclusion Criteria, and the Timeframe Covered

The inclusion criteria were as follows: review studies (narrative, systematic or meta-analysis) published between 2019 and 2022, specifically about ‘superfoods’ (or groups of foods) that can significantly reduce glycaemic levels in T2DM patients. The last 3 years were purposively covered to include more recent and updated information on the present topic. Other topics, and papers written in languages other than English, Portuguese, French, Spanish and Italian, were excluded.

### 2.2. Type of Study

A narrative review was conducted. The present narrative review was classified as suitable for solving/replying to the present study objectives and research questions, considering that (i) “different interventions for the same condition” and “different outcomes for the same intervention in the same condition” were summarised [10], and (ii) a high number of studies were covered, since only reviews were included/selected in the present narrative review.

### 2.3. Search Methodology and Data Extraction

The string(s) of keyword(s) were introduced to the databases or websites. Data were collected during January 2023 (last search 15 January 2023). Papers were conveniently selected in two steps, based on their compliance with the inclusion criteria. In the first step, (i) papers selected based on the abstract information were archived and (ii) the full text of all excluded papers was checked to validate (or invalidate) their exclusion. In the second step, the archived papers were fully analysed to validate their compliance with the inclusion criteria.

The extracted data were as follows: identified ‘superfood(s)’, year, country (i.e., country of affiliation of each author), type of review, total number of references of each selected paper, objective(s), examples of clinical evidence and main findings (effect on T2DM and other related diseases). The data collection was not intended to be exhaustive, i.e., only key findings were collected and summarised.

The same researcher conducted the search, selected the papers or other relevant documents/guidelines and summarised the key findings. Papers (or other documents) were selected based on their pertinence and contribution to the present topic.

### 2.4. Databases, Keywords and Data Presentation

The screened databases were as follows: PubMed, SciELO, DOAJ and Google Scholar. The screened keywords were [“type 2 diabetes” and (“food” or “diet” or “nutrition”) and (“glycaemia” or “glycemia”)]. Related keywords or MeSH terms were not screened.

The automatic PubMed fillers were activated as follows: meta-analysis, review and systematic review from 1 January 2019 to 30 December 2022. The keyword “review” was also included in the remaining screened databases (SciELO, DOAJ and Google Scholar). The search was carried out in all fields for all searched databases/resources.

A tabular format was used to present the study findings (see the Section 3).

### 2.5. Quality Assessment

SANRA (a scale for the quality assessment of narrative review articles) was strictly followed, based on: (1) the provision of a justification of the article’s importance for the readership; (2) a statement of concrete aims or the formulation of questions; (3) a description of the literature search; (4) referencing; (5) scientific reasoning; and (6) an appropriate presentation of the data [11]. Considering that the present work is a narrative review, the guidance for carrying out “overviews of reviews” was not strictly followed (please see study limitations) [12].

### 2.6. Previously Identified Reviews on the Present Topic

A review related to the present topic (to provide an overview of controlled human intervention studies with foods described as ‘superfoods‘ and their effects on metabolic syndrome parameters) was published in 2018. Overall, 113 intervention trials (up to April 2017) were identified in this review by van den Driessche, involving the following ‘superfoods’: blueberries (8 studies), cranberries (8), goji berries (3), strawberries (7), chili peppers (3), garlic (21), ginger (10), chia seed (5), flaxseed (22), quinoa (1), cocoa (16), maca (1), spirulina (7), wheatgrass (1), acai berries (0), hemp seed (0) and bee pollen (0). This review concluded that the evidence on the control of metabolic syndrome parameters due to the consumption of ‘superfoods’ was limited [6].

Considering that the present narrative review covers studies (narrative, systematic or meta-analysis) published between 2019 and 2022, there is no risk of duplication with the previous review by van den Driessche [6]. Moreover, the study objectives of the present narrative review and the review by van den Driessche [6] are different.

## 3. Results

### Selected Papers/Documents

Overall, from the five hundred eighty-six identified reviews, thirty reviews (100%) were conveniently selected and analysed in the present work: nine (30%) were systematic reviews, of which five (17%) were also meta-analyses. The main findings are presented in Table 1.

## 4. Discussion

### 4.1. Identified ‘Superfoods’ That Can Reduce Glycaemic Levels in Patients with Type 2 Diabetes Mellitus (T2DM)

The identified ‘superfoods’ that can reduce glycaemic levels in T2DM patients are summarised in Table 2.

#### 4.1.1. Foods with Polyphenols (e.g., Berries)

Polyphenols, and flavones such as luteolin (celery, parsley), quercetin (apple, broccoli), catechins (tea, chocolate), narigin (citrus fruit), genistein (soybean), cyanidin (grape, berries, onion), caffeic and ferulic acids (coffee, strawberries, wholegrain cereals), gallic acid (species, vinegar, grape), resveratrol (grape, wine), pinoresinol (cereals, sesame) and tannic acid (legumes, pomegranate) constitute bioactive compounds, which can (a) reduce carbohydrate digestion, triggered by the inhibition of intestinal enzymes (inhibition of α-glucosidase); (b) enhance glucose uptake (stimulating glucose transport into other functional tissue cells from the blood); (c) decrease gluconeogenesis; or (d) increase insulin secretion [17,18].

Besides a significant reduction in blood glucose, there was also an improvement in the lipidic profile and insulin resistance with these foods (e.g., with the daily consumption of coffee or tea). The daily consumption of naturally polyphenol-rich foods and beverages seems to provide a benefit in the control of cardiometabolic diseases. The diet should preferably contain different classes of polyphenols rather than a specific food or phenolic compound, since different classes of polyphenols seem to have a pleiotropic effect. For instance, the daily consumption of anthocyanidin-rich fruit (blueberries and apples) reduced the risk of T2DM by 23%. However, a positive association between the consumption of flavonoids and their subclasses and a reduced risk of T2DM was not found in all studies [18]. For instance, the light/moderate consumption of wine was associated with improved metabolic control in T2DM individuals due to the presence of non-alcoholic polyphenols, such as resveratrol [19]. However, the American Diabetes Association recommendations on alcohol intake should be followed: one drink a day for women and up to two per day for men (5 ounces of wine, a 12-ounce beer, or one and a half ounces of 80-proof spirits), because there are potential risks associated with alcohol consumption by T2DM patients, such as an increased hypoglycaemic risk, particularly when insulin and sulphonylureas are prescribed [43].

The bioactive compounds of berries in particular include powerful antioxidant anthocyanins (such as cyanidin and delphinidin), flavanols and phenolic acids (such as ellagitannins/ellagic acid), which can inhibit the enzymes amylase and glucosidase, preventing glucose absorption in the intestines (controlling postprandial glycaemia) and stimulating insulin secretion [14,15]. Berry anthocyanins enhance glucose uptake and metabolism through pAMPK/AMPK, GLUT-4 and SGLUT-1 activation; they also inhibit weight gain and pro-inflammatory responses, downregulating lipogenesis genes and pro-inflammatory cytokine production [16]. Additionally, anthocyanins support the positive modulation of plasma lipid levels, gut microbiota and endothelial function [13]. Findings between studies are not all in agreement with regard to the metabolic control effects in T2DM patients, which may be due to differences in the quantitative or qualitative anthocyanin intake. For instance, the profile of bioactive substances may vary with the product type or the variety and mixture of berries (e.g., wild blueberry, bilberry, cranberry, elderberry, raspberry seeds and strawberry). Additionally, health benefits may vary with the subjects’ baseline conditions (e.g., statistically significant alterations in glycaemia are unlikely in subjects with a normal glucose tolerance) [14]. Some studies propose that a moderate intake of berries (at least 50 mg anthocyanins, ⅓ cup of blueberries) is enough to mitigate the risk or to help in the metabolic control of T2DM, giving an example of the total anthocyanins (mg/100 g fresh) in blueberry highbush (387) and blueberry lowbush (487) [13,14]. However, other studies recommend a daily dose of 200 g to 400 g (70 kg; middle-aged person) [16].

#### 4.1.2. Fermented Dairy Products (with or without Vitamin D or Probiotic Supplementation)

Fermented dairy products not supplemented with vitamin D or probiotics, especially yoghurts, seem to be capable of improving glycaemic markers. They potentially help the metabolic control of T2DM through increased satiety (decreased food intake), improved insulin sensitivity and insulin resistance, increased glucose tolerance, an altered gut hormone response, changed gut microbiota and enhanced body fat reduction [20,24].

Yoghurt naturally includes lactic acid bacteria, which can promote favourable changes in gut microbiota, the amelioration of glycaemic control and insulin resistance and an increase in glucagon-like peptide 1 (GLP-1), with an anorexigenic effect. The superior bioavailability of amino acids and insulinotropic peptides and the bacterial biosynthesis of vitamins such as vitamin K2 can explain the improvement of insulin sensitivity through the vitamin K-dependent protein osteocalcin, anti-inflammatory properties and lipid-lowering effects [25]. Milk and dairy products are strong insulin secretagogues, as their intake causes acute hyperinsulinaemia, and they also contain bioactive peptides, calcium and B-complex vitamins, which support regulation of the microbiome and the metabolic control of diabetes [20,24]. However, high-fat dairy and animal proteins are associated with greater insulin resistance and lower HDL cholesterol [21].

Dairy products supplemented/fortified with vitamin D (100 IU to 28,000 IU vitamin/day) can potentially control T2DM by suppressing the activation of T cells and systematic inflammatory markers [22]. The supplementation of dairy products with probiotics can also have a positive impact on the metabolic control of T2DM patients, since these patients tend to have a microbial imbalance or dysbiosis in the gastrointestinal tract, as well as systemic low-grade inflammation. This dysbiosis and inflammation can support an increased ratio of Firmicutes to Bacteroidetes (two dominant phyla within the gastrointestinal tract), and a reduced presence of lactic acid, producing species such as *Lactobacillus*, *Bifidobacterium* and *Streptococcus*. For instance, “*Streptococcus thermophilus*, *Lactobacillus bulgaricus* and/or *Bifidobacterium lactis* administered for 6 to 12 weeks may be efficacious for improving glycaemic control in adults with T2DM” [26]. Thus, yoghurt enriched with probiotic bacteria can reinforce gut health, reduce inflammation, regulate appetite, improve immune responses and ameliorate the intestinal barrier function and lipid profiles. Probiotics support the modulation of the gut microbiome by increasing GLP-1 and stimulating the production of short-chain fatty acids (SCFAs), which also promote GLP-1 secretion in obese subjects [23].

The findings between studies with regard to the integration of probiotics in dairy products in the diet of T2DM are not in agreement. A significant impact on fasting insulin, HbA1c or plasma C-reactive protein (CRP) were not reported in some studies [23,44,45], in opposition to other studies that reported positive outputs in the metabolic parameters of T2DM patients [23,24,25,26]. For instance, one study indicated that multi-strain probiotics with 7 million to 100 billion colony-forming units of *Lactobacillus acidophilus*, *S. thermophilus*, *L. bulgaricus* and/or *B. lactis* administered for 6–12 weeks may effectively control glycaemia in T2DM [26]. Additionally, one study indicated that the probiotic supplementation of dairy matrices can reduce lipid concentrations and anthropometric parameters [23].

#### 4.1.3. Whole Cereals

The dietary fibre intake from whole grains decreases blood glucose, attenuates insulin responses and lowers inflammatory markers. Insoluble dietary fibres improve the whole-body insulin resistance after both a short-term and prolonged intake of cereal fibre. In general, fibre delays gastric emptying and decreases the intestinal absorption of glucose. Dietary fibre (both soluble and insoluble), phenolic compounds and other bioactive constituents may also decrease starch hydrolysis [27,28]. Whole grains are comprised of phenolic acids, such as hydroxybenzoic acids (p-hydroxybenzoic acid, gallic acid, vanillic acid and syringic acid) or hydroxycinnamic acids (ferulic acid, p-coumaric acid, caffeic acid and sinapic acid), which contribute to the overall antioxidant capacity (lowering oxidative stress), as well as having a high fibre content. Additionally, there is an inverse relationship between soluble dietary fibre intake and triglyceride levels [29]. The fermentation of fibre and resistant starch by microbiota in the large intestine leads to the production of SCFAs, which are linked to the secretion of gut hormones, glucose and lipid metabolism [27,28]. The consumption of whole grains therefore supports the regulation of intestinal microbiota, the synthesis of SCFAs, a reduction in transit time, the prevention of insulin resistance and the antioxidant activity of phenolic acids, which confer protection by binding carcinogens, as well as modulating the glycaemic response [29]. The consumption of whole grains also supports the control of long-term weight gain [27,28].

Whole grains should not be processed (e.g., barley, bulgur, farro, millet, quinoa, black rice, brown rice, red rice, wild rice, oatmeal, popcorn and whole-wheat flour), since this affects the dietary fibre composition and the release of phenolics. For instance, milling decreases the particle size and increases the surface area of cereals, which substantially increases starch hydrolysis and the glycaemic response [27].

#### 4.1.4. Nuts

In general, nuts present a lower glycaemic index (GI), an elevated content of fibre and non-sodium minerals (potassium and magnesium) and high levels of antioxidant and anti-inflammatory compounds. In addition to macronutrients, nuts also contain micronutrients, water-soluble vitamins such as folate, non-sodium minerals and phenolics (e.g., flavonoids, phenolic acids, stilbenes, coumarins, lignans and tannins, among others). The presence of minerals (e.g., potassium, calcium and magnesium) and vegetable protein (arginine) potentially contributes to the maintenance of glucose control. Additionally, fatty acids are the predominant components of nuts, which explains their ability to lower cholesterol (e.g., almonds, Brazil nuts, cashews, hazelnuts, macadamias, pecans, pine nuts, pistachios and walnuts) [31].

The intake of nuts is recommended as an alternative to the consumption of carbohydrates in individuals with T2DM because it improves glycaemic control and lipid risk factors [46]. Besides the potential to control glycaemia, the consumption of almonds has been related to weight control; decreased macronutrient bioavailability; reduced hunger; elevated satiety; increased resting energy expenditure; and a reduction of mean body mass, fat mass, diastolic blood pressure and low-density lipoprotein (LDL) cholesterol; as well as a possible improvement in colonic microbiota [32].

Additionally, the consumption of pistachios seems to result in better metabolic control (e.g., fasting blood glucose and Homeostatic Model Assessment of Insulin Resistance—HOMA-IR), with a simultaneous improvement in the cardiometabolic profile, endothelial dysfunction, inflammation and oxidative stress of patients with metabolic syndrome, as well as the possible amelioration of the lipid profile of obese patients. Thus, it seems that pistachio nuts may be an invaluable adjuvant therapy for the prevention and treatment of T2DM due to their high content of different types of antioxidants [33].

#### 4.1.5. Proteins (Especially Plant Protein-Rich Diets)

Diets rich in plant protein, white meat or fish protein improve the levels of total cholesterol in people with T2DM compared with diets rich in red meat protein. Fasting glucose levels were found to improve with a plant protein diet, and improvements in blood pressure and body weight were achieved with weight-loss diets: 23% to 32% of energy intake as protein (for up to one year) [34].

With regard to plant protein foods, including leguminous proteins, the consumption of both beans and chickpeas (e.g., hummus) leads to an improvement of glycaemic control and insulin sensitivity in T2DM patients [35,36,47]. Leguminous plants such as soybeans and pulses (dried beans, dried peas, chickpeas and lentils) can reduce insulin resistance and other related T2DM parameters (e.g., HOMA-IR), with higher evidence for soybeans and chickpeas than other leguminous plants [35]. For instance, the consumption of beans leads to a healthy gut microbial population and diversity (SCFAs are produced from the fermentation of the complex dietary fibre and resistant starches of beans, increasing butyrate production), and the gut microbiome supports the reduction of body weight and body fat, as well as improving insulin sensitivity [35]. The better metabolic control of glucose following the consumption of hummus (in terms of an improvement in glucose tolerance and the enhancement of insulin secretion) can be explained by its low sugar and high fibre/protein content, slow digestibility, slow rates of absorption, amino acid profile, soluble fibres (slower gastric emptying), high monounsaturated fatty acid (MUFA) and polyunsaturated fatty acid (PUFA) content, high levels of the resistant starch amylose which is digested and absorbed more slowly, and an increase in gut hormones such as GLP-1 and peptide YY because of the fermentation of amylose [36].

The consumption of the whey protein also supports the improvement of glycaemic control and insulin sensitivity in T2DM patients, which is explained by a rapid increase in bioactive peptides and amino acids (from the hydrolysation of the whey), increased insulin release and improved postprandial hyperglycaemia. Additionally, bioactive peptides activate the release of incretin hormones (gastric inhibitory polypeptide and GLP-1), which improve insulin resistance and inhibit dipeptidyl peptidase-IV, consequently reducing the degradation of gastric inhibitory polypeptide and GLP-1 [37]. Overall, whey supplementation was found to significantly decrease systolic blood pressure, diastolic blood pressure, HDL, waist circumference, triglycerides and fasting blood glucose in intervention groups when compared to control groups [48].

#### 4.1.6. Sunflower Seeds and Flaxseeds

The bioactive components of sunflower seeds (chlorogenic acid) and flaxseeds (secoisolariciresinol diglucosoid) can improve insulin resistance and insulin production [38].

#### 4.1.7. Cabbage and Lupin

According to epidemiological studies, crucifers such as cabbage may reduce the risk of T2DM due to the presence of bioactive compounds (e.g., vitamins, fibre, phenolic compounds, flavonoids, among others) which support the attenuation of oxidative stress, obesity, insulin resistance and hyperglycaemia [39]. However, according to the results of a recent systematic review and meta-analysis, brassica vegetables have no significant impact on the serum levels of triglycerides, LDL cholesterol, HDL cholesterol, fasting blood sugar and HbA1c in adults [49].

Lupin seems to be equally and possibly more effective among all the legumes in terms of the long-term health protection (e.g., satiety and glycaemic control, or serum lipid profile and blood pressure benefits) [40].

#### 4.1.8. Prickly Pear Cacti (*Opuntia* spp.) Cladodes

The high fibre content of prickly pear cladodes may explain the amelioration of gut microbiota, with reduced glucose absorption, improved glycaemic control and reduced glucose peaks [41]. Nopal (Opuntia ficus indica) seems to suppress the inflammatory effect of lipopolysaccharides, which are responsible for inducing insulin resistance and glucose intolerance. For instance, in patients with T2DM, a significantly lower area under a curve (AUC) for glucose (287 ± 30) was achieved in the high-soy-protein breakfast + nopal group compared to the high-soy-protein breakfast only group (443 ± 49) [50].

#### 4.1.9. Honey

The consumption of honey by T2DM patients may improve the glycaemic and lipid profile (although the effects of honey are acute/short term). These effects may be explained by its polyphenol content, the inhibition of α-amylase and α-glucosidase, a reduction of the inflammatory state and oxidative stress and the consequent protection from endothelial dysfunction and neurodegeneration [42]. The benefits of honey over isolated glucose as a sweetener were also confirmed in a study comparing the glycaemic effect of 75 g and 30 g of natural honey vs. 75 g of glucose in T2DM patients (*n* = 97), where results showed a mean rise in blood glucose of 85 mg/dL, 30 mg/dL and 170 mg/dL, respectively, (*p* < 0.005) after two hours [51].

### 4.2. Identified ‘Superfoods’ with the Potential to Reduce HbA1c in T2DM Patients

The number of reviews describing the potential reduction of HbA1c as a consequence of the consumption of certain ‘superfoods’ by T2DM patients is very limited and the results differ.

#### 4.2.1. Foods with Polyphenols (e.g., Berries)

A reduction in HbA1c was reported in some studies following the consumption of berries (e.g., 50 g of freeze-dried strawberry powder, equivalent to 500 g fresh strawberries each day, or 320 mg day^−1^ anthocyanin capsules—bilberry and blackcurrant), with a potential 0.20% reduction in HbA1c [16,52,53]. The consumption of some foods containing polyphenols (e.g., green tea, catechins or total polyphenols) also produced improvements in the HbA1c levels according to some studies, with a net change of −0.30% (95% CI: −0.37, −0.22) [18,54]. For instance, three months of supplementation with resveratrol (250 mg/once daily) significantly reduced the mean HbA1c (mmol/L) (mean ± SD: 9.99 ± 1.50 vs. 9.65 ± 1.54; *p* < 0.05), systolic blood pressure (mm Hg) (mean ± SD: 139.71 ± 16.10 vs. 127.92 ± 15.37; *p* < 0.05) and total cholesterol (mg/dL) (mean ± SD: 4.70 ± 0.90 vs. 4.33 ± 0.76; *p* < 0.05), i.e., resveratrol supplementation improved the metabolic control of T2DM patients [55]. Additionally, the consumption of total polyphenols was associated with lower HbA1c concentrations in an observational study with T2DM subjects (*n* = 3000): people with the highest intake of energy-adjusted polyphenols (upper tercile) had a more favourable cardiovascular risk factor profile compared to people with the lowest intake (lower tercile) (7.70% vs. 7.67% HbA1c) [56].

#### 4.2.2. Fermented Dairy Products

The consumption of fermented dairy products was associated with a reduction in HbA1c in some studies [20,24,26]. For instance, a daily intake of 250 mL kefir significantly lowered HbA1c levels (providing a mean reduction of 1.34% HbA1c, from 8.54% (±1.56) to 7.20% (±1.12)) compared with the control group, who only received metformin [57].

Additionally, yoghurt enriched with vitamin D or probiotics produced a significant reduction in HbA1c. Over 12 weeks, yoghurt containing 170 mg calcium and 500 IU vitamin D/250 mL produced a reduction in HbA1c of around 1%, from 8.7% ± 1.8 to 7.8% ± 1.3, with a much lower improvement in HbA1c in the group consuming yoghurt that was not enriched with vitamin D (from 8.9% ± 1.6 to 8.5% ± 1.6) [58]. Meanwhile, 120 g/day fermented milk containing *L. acidophilus* La-5 and *Bifidobacterium animalis* subsp lactis BB-12 (10^9^ colony-forming units/day, each) gave a mean reduction of −0.67% HbA1c, compared with a mean change of +0.31 HbA1c for the control group (conventional fermented milk) [59]. HbA1c (mmol/L) was also reduced from 7.61 ± 1.22 (before intervention) to 6.40 ± 1.91 (after intervention) at the end of an 8-week trial with fermented milk (kefir) enriched with probiotics (*n* = 60 T2DM patients) [60].

#### 4.2.3. Whole Cereals

The arabinoxylan in whole cereals can contribute to the reduction of fasting glucose and HbA1c in diabetic fat rats [27,61]. The consumption of whole cereals can also contribute to better insulin sensitivity, glucose homeostasis, the amelioration of lipid disorders, weight control, the regulation of gut microbiota and/or better antioxidant and anti-inflammatory effects [27,28,29]. An increased fibre intake was found to reduce HbA1c (mean difference −2.00 mmol/mol (−2.3%) (95% CI: −3.30 to −0.71) from 33 controlled trials), comparing a daily dietary fibre intake of 35 g with the average intake of 19 g (increasing the daily fibre intake by 15 g or up to 35 g) in prospective studies or controlled trials in adults with prediabetes, gestational diabetes, type 1 diabetes and T2DM [30].

Additionally, HbA1c levels were decreased in the whole-grain germinated brown rice group (T2DM patients) in a randomised control trial (RCT), but without significance [62]. A low-GI legume diet reduced HbA1c values by −0.5% (95% CI: −0.6% to −0.4%), while a high wheat fibre diet reduced HbA1c values by −0.3% (95% CI: −0.4% to −0.2%) (*n* = 121 participants with T2DM, randomised into two groups: (i) low-GI legume diet—increasing legume intake (such as beans, chickpeas and lentils) by at least 1 cup per day, and (ii) high wheat fibre diet—increasing insoluble fibre by the consumption of whole wheat products; 3 months) [63]. However, no positive effect of the consumption of whole grains was found on HOMA-IR, HbA1c or 2 h glucose in a meta-analysis of 32 RCTs [64], which may be explained by the fact that only four out of the thirty-two RCTs specifically enrolled T2DM patients.

#### 4.2.4. Nuts: Almonds and Pistachios

Overall, studies are not in agreement regarding the positive impact of nut consumption on the metabolic control of T2DM patients (e.g., fasting plasma glucose or HbA1c). Some studies describe a possible positive effect of some types of nuts, such as almonds and pistachios, on glycaemic control [31,32,33]. For instance, a reduction in HbA1c was reported following the consumption of almonds and pistachios in T2DM patients [32,33]. On the contrary, no favourable effects were found with regard to fasting blood glucose or HbA1c, as a consequence of consuming tree nuts or peanuts for 3 months [31,65], although only 14 out of the 40 RCTs specifically enrolled T2DM patients.

#### 4.2.5. Proteins (Especially Plant Protein-Rich Diets)

A moderately greater weight loss was attained with a higher protein hypocaloric diet in T2DM patients compared to a lower protein hypocaloric diet. As expected, significant improvements in HbA1c were achieved with weight loss diets, which were not influenced by higher protein diets [34]. Depending on the magnitude of weight loss, greater reductions in HbA1c can be expected in T2DM patients. For instance, a weight loss of ≥15% reduced HbA1c by an average of 1.2% (1 year of follow-up) [66]. Meanwhile, a low-GI diet (190 g (1 cup) of legumes (cooked beans, chickpeas or lentils) per day for 3 months) significantly improved HbA1c (−0.5% absolute) [63]. Additionally, the consumption of black bean pasta meals (different protein concentrations) reduced postprandial glycaemia and insulinaemia when compared to the control in adults (white bread) [67].

The consumption of whey protein significantly reduced HbA1c (*n* = 6 studies) (weighted mean difference: −0.15; 95% CI: −0.29, −0.01) [37]. Whey can be given immediately before a meal (e.g., 55 g whey protein preload, given 30 min before a meal), since it reduces the postprandial glycaemic response by over a third, which can be explained by an increase in the early postprandial plasma insulin and GLP-1 responses [68,69]. Thus, T2DM patients who have relatively well-controlled fasting glucose and a mild-to-moderate elevation of HbA1c may be more likely to benefit from the postprandial glucose lowering effects of whey protein [69].

#### 4.2.6. Flaxseed Supplementation

Flaxseed supplementation significantly reduced HbA1c (−0.19%; 95% CI: −0.38 to 0.00; *p* = 0.045; I^2^ = 12.8%) in participants with T2DM compared with the control group, but without an effect on fasting blood glucose (FBG) (−0.31 mmol/L; 95% CI: −0.86 to 0.25; *p* = 0.280; I^2^ = 82.9%). In particular, more significant reductions were reached for T2DM patients with a higher baseline level of HbA1c (e.g., HbA1c ≥ 7.0%) [70].

#### 4.2.7. Lupin

A daily dose of a 10 g *Lupinus mutabilis*-based snack, consumed 30 min before lunch, significantly reduced HbA1c in a group of patients with HA1c levels <8.0% (mean value (%) ± standard deviation: 6.5 ± 0.6 (baseline) and 6.3 ± 0.7 (28 weeks); around 0.2% reduction), with no significant improvements in the group of patients with HbA1c levels >8.0%. In addition, body weight and both systolic and diastolic blood pressure significantly decreased, while HDL cholesterol significantly increased by the end of the study period. However, the fasting glucose levels (mg/dL) increased from 116.3 ± 27.8 (baseline) to 134.1 ± 38.71 (28 weeks) [71]. In contrast, in another studies, no significant effects on glycaemic control or blood pressure were found with the regular consumption of lupin-enriched foods in T2DM patients [72].

#### 4.2.8. The Most Significant Reductions in HbA1c

**Figure 1:**
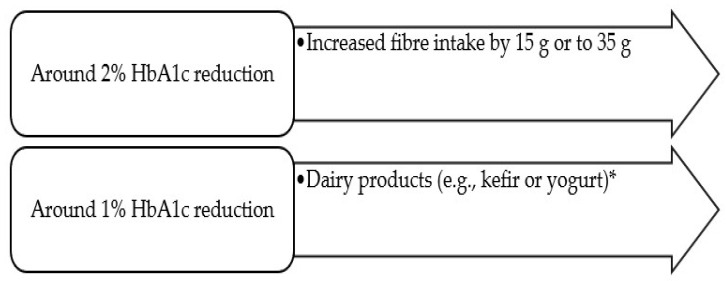
‘Superfoods’ related to significant HbA1c reductions (≥1%)* Especially, dairy products enriched with vitamin D or probiotics.

Overall, the most significant reported reductions in HbA1c were achieved through (i) the consumption of dairy products (e.g., kefir or yoghurt) (HbA1c reduction of around 1%), with more significant reductions appearing to be attained with dairy products enriched with vitamin D or probiotics compared to non-enriched dairy products [58,59]; (ii) an increased fibre intake (by 15 g or up to 35 g) (HbA1c reduction of around 2%) [30] (Figure 1); or (iii) diets with weight reduction: a weight loss ≥15% reduced HbA1c by an average of 1.2% (1 year of follow-up) [66]. Thus, both dairy products, such as kefir or yoghurt (with or without vitamin D and/or probiotics) and an increased fibre intake (by 15 g or up to 35 g) [30,58,59] can be used as nutraceuticals, i.e., “a food (or part of a food) that provides medical or health benefits, including the prevention and/or treatment of a disease” [73].

### 4.3. New Proposed International Guidance on the Consumption of Identified ‘Superfoods’ by T2DM Patients

A proper medical nutrition therapy for T2DM patients may be more easily achieved and/or maintained with the concomitant use of ‘superfoods’, since they can improve or maintain metabolic control (glucose and lipid profiles, body mass weight, blood pressure and anti-inflammatory markers, among others). Thus, the following recommendations are proposed:

- The isolated consumption of ‘superfoods’ should not be used to substitute a proper and successful diet or exercise plan for T2DM patients. The adoption of a certain diet, such as a Mediterranean-style, low-fat or low-carbohydrate diet, seems to be more relevant than the isolated consumption of ‘superfoods’ by T2DM patients, since it has been demonstrated that a successful medical nutrition therapy plan per se can reach a similar or greater reduction in HbA1c than medication for T2DM [8].
- It is likely that the goals of a proper medical nutrition therapy for T2DM patients may be more easily achieved and/or maintained with the concomitant use of ‘superfoods’, since some ‘superfoods’ are likely to improve or maintain metabolic control (glucose and lipid profiles, body mass weight, blood pressure and anti-inflammatory markers, among others).
- ‘Superfoods’ should preferably be integrated into the diet plan of T2DM patients with the involvement of a nutritionist. For instance, ‘superfoods’ can be used to substitute foods from the same group of the food wheel, respecting the principles of a diversified and rational nutritional plan.
- Metabolic supervision should be carried out in the months before and after the introduction of ‘superfoods’ into the diet of T2DM patients to identify and quantify eventual ameliorations.
- ‘Superfoods’ should be consumed in the right doses (quantitatively and qualitatively) to ensure that their bioactive properties are achieved, particularly given that ‘superfoods’ tend to be more expensive than other foods.
- Less-controlled T2DM patients can benefit more from the inclusion of ‘superfoods’ in their diet than more-controlled T2DM patients.
- More significant HbA1c reductions seem to be achieved with the consumption of dairy products (giving a reduction of around 1%, with or without the enrichment of vitamin D and probiotics, according to diverse studies) [57,58,60] and with fibre supplementation (based on the comparison between a daily dietary fibre intake of 35 g and the average intake of 19 g: increasing the daily fibre intake by 15 g or up to 35 g resulted in a reduction of −2.00 mmol/mol (−2.3%) HbaA1c) [30]. Thus, these ‘superfoods’ are recommended in the diet of T2DM patients, although this does not exclude the need for metabolic control (before and after any alteration in the diet plan), since results are not in agreement between studies.
- According to some studies, better results are achieved with the consumption of dairy products supplemented with vitamin D (++) or probiotics (+) compared to unenriched dairy products [58,59].
- High-fat dairy products are not recommended [21,23]. The consumption of cheese can result in a 5 to 24% increase in the risk of developing T2DM according to some prospective cohort studies [23]. Thus, the consumption of high-fat dairy products should be limited (or eliminated) in the diets of T2DM patients.
- Refined grain products should be substituted by whole grain foods, since diverse studies support an improvement in the metabolic metabolism and lipid profile of T2DM patients, a decreased risk of T2DM and a possible decreased risk of colon cancer, fatal coronary heart disease (CHD) and cardiovascular disease (CVD) mortality with the consumption of whole grain foods [28].
- Different classes of foods with polyphenols (e.g., berries) are recommended rather than a specific food due to a likely pleiotropic effect, i.e., a potential contribution towards the regulation of glycaemic and lipidic metabolism (increased HDL cholesterol and decreased LDL cholesterol), blood pressure control and anti-obesity, as well as an improvement in the anti-inflammatory and oxidative stress plasma markers [14,16].
- Proteins (especially plant protein-rich diets), such as beans/chickpeas, other leguminous plants (e.g., soybeans) and other proteins, such as whey protein, are recommended in the regular diet of T2DM patients, since they support an improvement in glycaemic control and insulin sensitivity [34,35,36].
- Nuts (without added salt or sugar) are recommended, especially to control the lipidic profile (total cholesterol, LDL and triacylglycerols) [31].
- Almonds and pistachios, in particular, can reduce fasting plasma glucose, including HbA1c, although results are not conclusive [32,33].
- Nuts seem to be an adequate alternative to the consumption of carbohydrates, with improved glycaemic control and lipid risk factors in individuals with T2DM [46].
- Other ‘superfoods’, such as sunflower seeds, flaxseeds, cabbage, lupin, prickly pear cacti (*Opuntia* spp.) cladodes and honey (short term), may also improve the metabolic control of T2DM patients, but further studies are recommended.

### 4.4. Strengths and Weaknesses of the Present Narrative Review

Overall, the 30 selected reviews covered 3234 references (sum of all cited references), and 31% of the selected works were systematic reviews, which seems to confirm the quality, robustness and relevance of the present review. The evidence emerging from the present narrative review provides up-to-date information on the use of ‘superfoods’ in T2DM patients, compared to the findings of van den Driessche et al. or to some of the international recommendations [5,6], for example (see Introduction and Methods). New updated recommendations on the use of ‘superfoods’ in the diet of T2DM patients are also proposed in this narrative review.

On the other hand, diverse limitations can be highlighted. Since the present work is a narrative review, this review was only carried out by one author, which may have introduced bias with regard to the data collection or interpretation, and a study protocol was not previously registered. In addition, papers were conveniently selected based on their compliance with the inclusion criteria, and the number of searched keywords and databases was limited [12]. For instance, the screened keywords were [“type 2 diabetes” and (“food” or “diet” or “nutrition”) and (“glycaemia” or “glycemia”)], and additional keywords or related MeSH terms were not screened. However, these issues are not mandatorily addressed in a narrative review. Additionally, the present work does not cover all ‘superfoods’ with potential relevance in the diet of T2DM patients.

### 4.5. Future Research

An “overview of reviews” should be carried out according to the guidelines of Gates et al. [12], with the involvement of more experts, including endocrinologists, diabetologists and nutritionists. More clinical studies should also be performed to determine the exact types and amounts of ‘superfoods’ that should be recommended to produce cardiometabolic benefits, depending on the BMI or the level of metabolic control of T2DM patients, for instance. More evidence should be generated in relation to the impact of consuming ‘superfoods’ on the significant reduction of HbA1c, since study findings are not in agreement, and the potential reduction of HbA1c only seems to be associated with the consumption of some ‘superfoods’. The impact of ‘superfoods’ on the glycaemic control of patients medicated with anti-diabetics should also be evaluated.

## 5. Conclusions

Overall, a diverse range of ‘superfoods’ was identified with possible metabolic benefits in the diet of T2DM patients (e.g., improved glycaemic and lipidic profile): foods with polyphenols (e.g., berries), fermented dairy products, whole cereals/grains, nuts (e.g., almonds and pistachios), proteins (especially diet plant proteins, Leguminosae and whey protein), sunflower seeds and flax seeds, cabbage, lupin, prickly pear cacti (*Opuntia* spp.) cladodes and honey (especially short-term use).

However, a limited number of ‘superfoods’ showed the potential to reduce HbA1c (reductions of 1% to 2%): fermented dairy products (with or without supplementation with vitamin D or probiotics) and fibre (increasing the daily fibre intake by 15 g or up to 35 g). In particular, the daily intake of dairy products, especially yoghurts, demonstrated a positive impact on the metabolic control of T2DM, such as a reduction in fasting plasma glucose and Hb1Ac, with better results for low fat yoghurts (e.g., kefir) supplemented with vitamin D (++) or probiotics (+) compared to unenriched yoghurts, according to some studies. These foods can therefore be used as nutraceuticals.

Diets associated with weight loss (e.g., low-GI or plant diets, especially high-protein diets) may be associated with extensive HbA1c reductions (e.g., a weight loss ≥ 15% reduced HbA1c by an average of 1.2%; 1 year of follow-up) [66]. The remaining identified ‘superfoods’ with the potential to reduce HbA1c (by around −0.1% to −0.3%) were as follows: foods with polyphenols (e.g., berries), nuts (e.g., almonds and pistachios), cooked beans, chickpeas, whey protein, flax seeds and lupin.

Based on these study findings, a set of new international recommendations has been proposed regarding the consumption of the identified ‘superfoods’ by T2DM patients. ‘Superfoods’ should not be used to substitute a rational diet and exercise plan, and they should preferably be included in the diet of T2DM patients by a nutritionist, or glycaemia should be monitored before and after the introduction of ‘superfoods’ in the diet of T2DM diabetic patients, with the aim of identifying the potential short- and long-term benefits.

## Figures and Tables

**Table 1:** Findings about the consumption of ‘superfoods’ on the metabolic control of TD2M.

Food	Year and Country/Type of Review (Total Number of References)	Objective(s)	Examples of Clinical Evidence	Main Findings: Effect on Type 2 Diabetes and Other Related Diseases
Blueberries [13]	Canada, United Kingdom, USA, Spain Narrative review (*n* = 198 references)	*To review the role of blueberries in cardiometabolic health.*	Biomarkers: improved insulin sensitivity, plasma lipid profiles and reduced plasma markers of oxidative stress (for both short- and long-term interventions).	A reduced risk of type 2 diabetes, cardiovascular disease, weight gain and death were reported. As well, neuroprotection and vision (in less extended) benefits.
Bilberry [14]	China Narrative review (*n* = 100 references)	*To review the potential effects of bilberry supplementation on metabolic and cardiovascular risk factors.*	Anti-inflammatory and hypoglycemic effects (e.g., decreased postprandial glycaemia and insulin level). Improvement of hyperlipidaemia: increased HDL cholesterol and decreased LDL cholesterol. Reduced blood pressure.	Bilberry supplementation can present a positive effect on metabolic and cardiovascular risk factors.
Red Raspberry (RR) [15]	USA Narrative review (*n* = 60 references)	*To investigate the potential metabolic benefits of dietary red raspberry in individuals with T2DM and prediabetes.*	The consumption of 250 g of frozen RR with a high-fat breakfast meal reduced postprandial plasma glucose levels and area under the curve (AUC): individuals with either T2DM or prediabetes and overweight or obesity (2 RCTs). Reduction of low-density lipoprotein cholesterol (LDL-c) (32 RCT).	Improvements in glycaemia profile and insulin sensitivity, adiposity, lipid profiles, ectopic lipid accumulation, inflammation, oxidative stress and cardiac health.
Berries * [16]	Poland Narrative review, with application of PRISMA flow diagram (*n* = 329 references)	*To summarise both clinical and non-clinical findings that the consumption of berries, berry extracts, purified compounds, juices, jams, jellies and other berry byproducts aided in the prevention or otherwise management of T2DM.*	Glucose-lowering and insulin sensitivity improvements. Lower of dyslipidaemia markers. Anti-obesity. Reduced oxidative stress markers. Improvement in endothelial function of subjects with metabolic syndrome. Anti-inflammatory and anti-hypertensive. Daily recommended dose: 200 to 400 g (70 kg; middle-aged person). Reduction of HbA1c was reported with berries in some studies (strawberries, raspberry and Acai berries).	The consumption of berry products is effective to prevent and control metabolic hyperglycaemic and hyperlipidaemic conditions.
Food with polyphenols [17]	China Narrative review (*n* = 159 references)	*To analyse how dietary polyphenols affect starch digestion.*	Polyphenols can directly inhibit key digestive enzymes (α-glucosidase) and bind with starch; bounded polyphenols are still able to show the inhibitory activity against the enzymes (in vitro studies).	A better postprandial hyperglycaemia profile by polyphenols may be explained by both inhibited starch digestion and impact on glucose transport.
Foods with polyphenols * [18]	Italy Narrative review (*n* = 136 references)	*To discuss the effect of polyphenol/phenolic compounds on the main cardiometabolic risk factors.*	Epidemiological studies: polyphenol-rich diets seem to benefit the prevention of T2DM risk. Medium-term clinical trials (6–8 weeks) improve blood glucose, lipids and blood pressure (individuals with and without T2DM). Best results for long-term RCTs, with polyphenol-rich foods and beverages. Improvement of HbA1c (green tea, catechins or total polyphenols intake) in some trials.	Different polyphenol food subclasses seem to present a pleiotropic effect on cardiometabolic risk factors. Fasting glucose, insulin or HbA1c can be improved according to some trials.
Grapes and their derivatives (wine) [19]	Italy, Germany, Australia, France, Greece Narrative review (*n* = 62 references)	*To confirm whether there is a difference between alcoholic beverages in inducing beneficial health effects in T2DM individuals and whether the consumption of alcoholic beverages can be included in the daily diet of diabetics.*	A reduced risk of T2DM with light to moderate wine consumption (ten studies).	Wine was the alcoholic beverage with the most favourable outcomes. Light/moderate wine consumption is associated with improved metabolic control in T2DM individuals.
Short- and long-term intake of fermented dairy products * [20]	Canada, UAE, USA Narrative review (*n* = 47)	*To evaluate the evidence from a cross-sectional analysis of longitudinal studies and human and animal experimental trials to further understand the current knowledge linking short- and long-term consumption of fermented dairy products to T2DM.*	Cohort studies: protective effect of fermented dairy products on the prevention and control of T2DM, especially with yoghurt’s potential to decrease insulin resistance and improve glycemic control: improved glucose tolerance, fasting blood sugar, HbA1c (1.2% to 2% reduction) and 2 h postprandial glucose.	Higher intake of fermented dairy products may decrease the risk of developing T2DM in the long term. In the short term, improvements in the glycaemic control markers were also achieved. Yoghurt was the most consistent food protecting against T2DM.
Dairy products and plant proteins (e.g., legumes and soy) [21]	Italy and USA Systematic review (*n* = 50)	*To evaluate the ideal protein quality and quantity and the dietary composition for the prevention and metabolic control of T2DM.*	RR (relative risk) 95% CI of T2DM: 0.89 (0.84–0.94) total dairy products, 0.87 (0.78–0.96) whole milk, 0.83 (0.70–0.98) yoghurt and 0.74 (0.59–0.93) soy (women).	Higher intake of plant protein and dairy products is associated with a modestly reduced risk of T2DM. Red meat, processed protein foods and high-fat dairy products could have negative effects in the long term.
Both dairy and not-dairy products fortified with vitamin D * [22]	Iran and UK Systematic review and meta-analyses (*n* = 48)	*To evaluate effects of Vitamin D fortification on indices of glycaemic control.*	In total, 11 RCTs; the impact of enriched food with vitamin D (100 IU to 28,000 IU vitamin/day) on fasting serum glucose in diabetics was significant (mean difference: −2.772, *p* = 0.041, and 95% CI: −5.435 to −0.109) as well as on serum insulin (mean difference: −2.937; 95% CI:−4.695 to −1.178).	Vitamin D fortification of dairy products leads to an improvement in Homeostasis Model Assessment of Insulin Resistance (HOMA-IR), fasting plasma glucose and HbA1C.
Fermented dairy products (FDFs) and probiotic supplementation * [23]	Spain Systematic review and meta-analysis (*n* = 107)	*To study the relation between the regular consumption of FDFs and cardiometabolic diseases (CMD) risk factors (assessed by prospective cohort studies–PCSs), and the effect of probiotic supplementation added into a dairy matrix on CMD parameters (evaluated by RCTs).*	In total, 20 PCSs and 52 RCTs. Probiotic intake in capsule/powder displayed a significant reduction on HbA1c changes. However, the effects of probiotic supplementation into a dairy matrix on diabetic parameters in T2DM subjects did not show significant results, regarding the alterations of fasting insulin, HbA1c and plasma CRP.	Yoghurt intake: reduced risk of T2DM and metabolic syndrome development. Fermented milk: reduced cardiovascular risk. Probiotic supplementation added into dairy matrices: can reduce lipid concentrations and anthropometric parameters. Probiotic capsule/powder supplementation: can favour T2DM management and reduce anthropometric parameters.
Yoghurt (enriched with probiotics) * [24]	Greece Narrative review (*n* = 97)	*To present the RCTs which have been conducted in the last decade in patients with T2DM.*	Significant reductions of HbA1 were reported in some trials. However, the number of trials is limited. Enrichment of yoghurts with vitamin D, calcium and probiotics (e.g., L. acidophilus and B. lactis during a short period of 4–12 weeks). Yoghurt enriched in flaxseed or complex B vitamins may have impact on glycaemia control.	The daily intake of yoghurt, especially when enriched with probiotics, vitamin D and calcium has a positive impact on the metabolic control of diabetic patients.
Fermented dairy (FD) foods rich in probiotics (e.g., cheese and yoghurt) [25]	Spain Narrative review (*n* = 76)	*To evaluate the relationship between the FD products: yoghurt and cheese, and cardiometabolic risk factors obtained from meta-analyses, systematic reviews of prospective cohort studies (PCSs).*	In all, 13 PCSs supported a potential protective role of yoghurt consumption and prevention of T2DM. Reduction in the risk of T2DM: 14% with a yoghurt intake of 80 g/d compared with no yoghurt intake and 22% with a yoghurt intake of 200 g/d (meta-analyses of PCSs).	Intake of yoghurt seems to be associated with a lower risk of developing type 2 diabetes. A lower risk of developing stroke and cardiovascular disease may be explained by the total consumption of FD.
Probiotics * [26] Dairy medium (e.g., goat’s milk, kefir and yoghurt); enriched with multiple probiotic strains).	USA Systematic review (*n* = 65)	*To evaluate nine randomised controlled trials that tested the effects of probiotics on glycaemic outcomes and insulin resistance.*	Nine randomised controlled trials. Improvement of insulin resistance; reduction of FPG, A1c, FPI and HOMA-IR with probiotic supplementation among subjects withT2DM.	Probiotic (dairy products and capsules) supplementation seems to favour metabolic control in adults with T2DM.
Whole cereals (e.g., wheat, rice, maize, barley, sorghum, millet, oat, rye, buckwheat) [27]	China, USA Narrative review (*n* = 384)	*To highlight recent findings on the influences of both bioactive constituents and processing on the anti-diabetic effects and physiological properties of cereals.*	The bioactive components of whole cereals, such as resistant starch, dietary fibre or β-glucan can contribute to reducing postprandial serum glucose and increasing insulin sensitivity.	Reduced risk of T2DM, with improvement of metabolic metabolism and lipidic profile.
Whole grains [28]	Italy, UK, Netherlands, Australia Systematic review (*n* = 75)	*To systematically review current evidence on whole grain consumption and various health outcomes provided from meta-analyses of observational studies.*	Whole grain consumption improves acute postprandial glucose and insulin homeostasis compared to similar refined foods in healthy subjects (meta-analysis of RCTs).	Strongest evidence: a decreased risk of T2DM and colorectal cancer with higher compared to lower dietary intake of whole grains. Possible: decreased risk of colon cancer, fatal coronary heart disease and cardiovascular disease (CVD) mortality.
Whole grains’ phenolic acids and dietary fibre (wheat, barley, oats, rice and buckwheat) [29]	China Narrative review (*n* = 172)	*To review the existing literature on the linkages between the consumption of whole grains and the development of the following chronic non-communicable diseases: CVDs, obesity, T2DM and cancer.*	Clinical and epidemiological studies support a positive association between the consumption of whole grains, phenolic acids and dietary fibres with a lower risk of disease. For instance, three or more servings of whole grains/day (20–30% lower risk of diseases).	The consumption of whole grains reduces the risk of CVDs, obesity, T2DM and cancer, with improved glycaemic control, and prevention of insulin resistance.
Dietary fibre and whole grains * [30]	New Zealand Systematic review and meta-analyses (*n* = 97)	*To evaluate the role of high-fibre diets on mortality and increasing fibre intake on glycaemic control and other cardiometabolic risk factors of adults with prediabetes or diabetes.*	Higher intakes of dietary fibre produced a reduced risk of premature mortality (prospective cohort studies) and improvement of glycaemic control and other risk factors for cardiovascular disease, such as cholesterol levels, HbA1c and body weight (controlled trials).	Improvement of glycaemic control and other risk factors for cardiovascular disease, such as cholesterol levels and body weight. Reduction of premature mortality.
Nuts (e.g., almonds, Brazil nuts, cashews, hazelnuts, macadamias, pecans, pine nuts, pistachios and walnuts) [31]	Turkey and Spain Narrative review (*n* = 64)	*To provide an overview of recent findings on bioactive constituents, health claims and health benefits of nuts and dried fruits.*	Reduction: total cholesterol; LDL-cholesterol; and triacylglycerols. No change: HDL and inflammation. No change/slight reduction: body weight and visceral adiposity (evidence from many studies). Epidemiologic studies (evidence from many studies): reduction of the risk of cardiovascular disease; coronary heart disease; cancer and all-cause mortality.	Nuts (e.g., 30 or 42.5 g/day without added salt or sugars) are potentially relevant to reduce total cholesterol, LDL and triacylglycerols (cardiovascular and coronary heart disease). The evidence regarding diabetes control improvement, including insulin sensitivity, is limited in clinical studies. The consumption of dried fruits presents similar benefits, although the level of evidence is more limited than for nuts.
Almond [32]	USA Comprehensive review (*n* = 131)	*To provide an in-depth analysis of the effect of almonds on weight measures, metabolic health biomarkers and outcomes and the colonic microbiota.*	RCTs (randomised controlled trials) are not consensual, regarding the benefits for glycaemic and HbA1s, with some studies showing positive results (and others not).	Consistent improvement of blood lipid profiles and modest reductions in blood pressure, but inconsistent and/or insignificant beneficial effects of glycaemic control, and HbA1c.
Pistachio [33]	Iran, Australia A systematic review and meta-analysis (*n* = 44)	*To evaluate the effects of pistachio nuts on glycaemic control and insulin sensitivity in patients with T2DM, prediabetes and metabolic syndrome.*	In all, 6 RCT; significant reduction in fasting blood glucose (FBG) and homeostasis model assessment of insulin resistance, but no significant improvement was observed with regard to hemoglobin A1c and fasting plasma insulin level.	Pistachio nuts might cause a significant reduction in fasting blood glucose and HOMA-IR, although HbA1c and fasting plasma insulin might not significantly improve.
Proteins * (especially plant protein rich diets) [34]	Germany, Australia, Finland, Sweden, Norway, Greece, Canada, USA, Croatia, Spain, Denmark Narrative review (*n* = 53)	*To review the literature regarding protein intakes.*	For instance, plant protein diet containing 65% plant protein, including 30% soy protein and 35% animal protein vs. control diet containing 30% plant and 70% animal protein (*n* = 41 participants); improvement in fasting glucose. Weight loss reduced HbA1c in all studies, although without significant differences between groups (high vs. low protein groups).	Protein rich diets, especially plant protein rich diets, improve the total levels of cholesterol and fasting blood glucose levels. High protein hypocaloric diets may moderately favour weight loss, when compared to lower protein hypocaloric diets, with a possible improvement in HbA1c and systolic and diastolic blood pressure.
Beans [35]	USA Narrative review (*n* = 96)	*To provide an overview of the benefits of plant-based eating, with a concise focus on the nutritional properties unique to dry beans and their connection to improved health parameters of obesity including cardiovascular, metabolic, gastrointestinal gut health and low-grade inflammation.*	Coronary heart disease (CHD) risk reduction and improved glycaemic control in T2DM patients.	The inclusion of beans in a plant-based diet presents a protective cardiovascular, metabolic and colon effect; improvement of obesity; management of immune-related disease and low-grade inflammation.
Chickpeas/Hummus (i.e., primarily chickpeas and tahini) [36]	USA Comprehensive review (*n* = 86)	*To provide a comprehensive review of the scientific evidence examining the effects of acute and long-term consumption of hummus and hummus ingredients on diet quality and risk factors related to T2DM, cardiovascular disease and obesity.*	Healthy adults (*n* = 10) consumed hummus (28, 56, 112, 259 g servings) with and without white bread vs. white bread alone: postprandial glucose AUC was lower for hummus alone < hummus and white bread < white bread alone. The 28 and 112 g servings of hummus also resulted in lower insulin AUC than the white bread serving (both, *p* < 0.05), but not with the 259 g serving (*p* > 0.05).	Improvement of postprandial glycaemic control, fasting lipids, appetite control and daily food intake with hummus compared to other consumed foods, such as white bread. Tahini showed a little impact on glucose control.
Whey protein * [37]	Iran A systematic review and meta-analysis (*n* = 54)	*To assess the effects of whey protein on serum lipoproteins and glycaemic status in patients with metabolic syndrome (MetS) and related disorders.*	Twenty-two studies: significant reduction of HbA1c, insulin and HOMA-IR, triglycerides levels, total cholesterol, LDL-cholesterol levels and total cholesterol/HDL cholesterol ratio.	The consumption of whey protein can improve HbA1c, insulin, HOMA-IR, triglycerides, total cholesterol, LDL cholesterol and total/HDL-cholesterol ratio in patients with MetS and related disorders.
Sunflower seeds and flax seeds [38]	Pakistan Narrative review (*n* = 21)	*To determine the effect of sunflower seeds and flax seeds on T2DM.*	For instance, 20 g/day of flaxseeds for three months reduced FPG concentrations, insulin resistance and insulin sensitivity.	Sunflower and flax seeds consumption can reduce glucose levels with better insulin resistance and improved insulin production.
Cabbage [39]	Mexico Narrative review (*n* = 201)	*To analyse the effects of cabbage, and its bioactive compounds, on glucose homeostasis.*	Improvement of glucose levels and oxidative stress and hypolipaemic markers.	Cabbage consumption can regulate glucose homeostasis.
Lupin * [40]	Australia, Systematic review (*n* = 37)	*To investigate the effects of lupin on a range of health outcome measures.*	There were 21 studies (998 participants): statistically significant decrease of glucose AUC in some studies. Benefits, such as satiety, glycaemic control or improved serum lipid profile and blood pressure were better with whole lupin.	The benefits on glycaemic control and serum lipid profile were moderate.
Prickly pear cacti (Opuntia spp.) cladodes [41]	Australia, UK, Greece, Italy Narrative review (*n* = 73)	*To summarise the latest findings on the consumption of the prickly pear (PP; Opuntia spp.) cladode as a potential nutritional tool for the management of hyperglycaemia.*	Reduction of glucose levels after prickly pear cladodes’ consumption (mainly acute studies).	Prickly pear cladodes show potential hypoglycaemic effects.
Honey [42]	Italy Narrative review (*n* = 112)	*To summarise the current literature concerning the beneficial effects of honey in the management of the obesity-related dysfunctions, including neurodegeneration.*	Honey significantly reduced plasma glucose concentration (especially, in the short term). However, plasma levels of haemoglobin A1c increased, after 8-week honey consumption in one clinical study.	Honey seems to improve glycaemic control and lipidic profile (honey acute effects/short term). Long-term clinical studies are limited.

* Reported potential to reduce HbA1c.

**Table 2:** ‘Superfoods’ that can reduce glycaemic level in type 2 diabetic patients.

‘Superfoods’	Examples	Reference(s)
Foods with polyphenols	Berries, tea, coffee, wine or grapes	[13,14,15,16,17,18,19]
Fermented dairy products	Yoghurt (fortified or not with vitamin D or probiotics)	[20,21,22,23,24,25,26]
Whole cereals/grains	Mixed grains, barley or oatmeal	[27,28,29,30]
Nuts	Almonds, Brazil nuts, cashews, hazelnuts, macadamias, pecans, pine nuts, pistachios and walnuts	[31,32,33]
Proteins (especially, plant protein-rich diets)	Chickpeas (e.g., hummus), beans or other proteins–whey protein	[34,35,36,37]
Other foods	Sunflower seeds and flax seeds; cabbage; lupin, prickly pear cacti (Opuntia spp.) cladodes and honey (especially for short-term use)	[38,39,40,41,42]

## Data Availability

All synthesized and generated data are available within the present narrative review.
